# Hydrogen improves the efficacy of tetrandrine in the treatment of silicosis by inhibiting vascular endothelial mesenchymal transition caused by oxidative stress

**DOI:** 10.3389/fbioe.2025.1668524

**Published:** 2026-01-05

**Authors:** Kang Xiao, Ming Li, Yingwen Mu, Yuxin Sun, Sirui Wang, Shangya Chen, Jiazi Ma, Mao Cao, Yong Yang, Hua Shao, Xuansheng Ding, Guanqun Cui, Zhongjun Du

**Affiliations:** 1 School of Basic Medicine and Clinical Pharmacy, China Pharmaceutical University, Nanjing, Jiangsu, China; 2 Shandong Academy of Occupational Health and Occupational Medicine, Shandong First Medical University & Shandong Academy of Medical Sciences, Ji’nan, Shandong, China; 3 Institute of Toxicology, School of Public Health, Cheeloo College of Medicine, Shandong University, Ji’nan, Shandong, China; 4 College of Clinical and Basic Medical Sciences, Shandong First Medical University & Shandong Academy of Medical Sciences, Ji’nan, Shandong, China; 5 Department of Respiratory Medicine, Children’s Hospital Affiliated to Shandong University, Ji’nan, Shandong, China; 6 Department of Occupational Disease, Harbin Second Hospital, Harbin, Heilongjiang, China

**Keywords:** hydrogen, tetrandrine, oxidative stress, EndMT, pulmonary fibrosis

## Abstract

**Objective:**

Silicosis, a lung disease associated with occupational exposure. Tetrandrine has been approved for the treatment of silicosis in China, but it still cannot be cured. This study aims to investigate the reasons behind the low concentration of tetrandrine (Tet) in lung tissue and propose a treatment plan.

**Methods:**

We first established a silicosis mouse and employed a combination of histological examination, Western blot analysis, immunofluorescence, and single-cell RNA sequencing to clarify the relationship between oxidative stress vascular endothelial mesenchymal transition (EndMT), and Tet concentration in lung tissue.

**Results:**

The study indicated that there is excessive activation of OS and EndMT in silicosis while concurrently reducing Tet concentration in lung tissue (from 94.8 ± 10.4 ng/mg to 54 ± 6.2 ng/mg). Furthermore, combined inhalation of hydrogen (H_2_) improved both the severity of silicosis and Tet concentration in lung tissue (from 50 ng/mg to 80 ng/mg). The proposed mechanism suggests that H_2_ inhibits the release of amyloid precursor protein (APP) in apoptotic alveolar macrophages. Additionally, the interaction between APP and CD74 in vascular endothelial cells was diminished, thereby inhibiting biological processes associated with endothelial mesenchymal transition, alleviating pulmonary vascular stenosis, and enhancing the concentration of therapeutic agents in lung tissue.

**Conclusion:**

Hydrogen can improve the tissue concentration of tetrandrine by anti-OS-induced EndMT.

## Graphical Abstract

**Figure:**
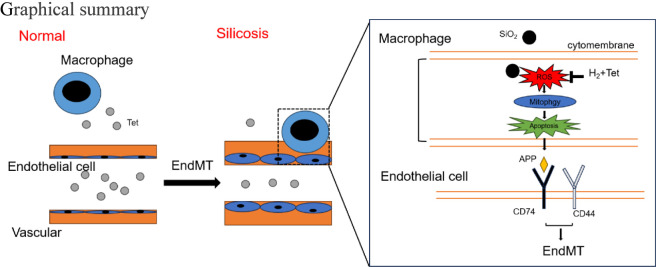

## Introduction

Pulmonary fibrosis caused by silicon dioxide (SiO_2_) is a significant occupational disease globally (Greenberg et al., 2007; Hoy and Chambers, 2020). In 2019, there were 138,971 new cases of silicosis worldwide, an increase of 64.61% compared with 1990 (84,426 cases) (Huang et al., 2024). Due to the limited effectiveness of treatment methods, silicosis has considerable negative impacts on patients and imposes a substantial economic burden on society.

Currently, significant progress has been made in the research on silicosis. Silicon-induced lung injury may result from multiple interacting pathological mechanisms, including the damage caused to macrophages by the phagocytosis of SiO_2_, the reactive oxygen species (ROS) production, mitophagy and apoptosis (Adamcakova and Mokra, 2021). According to global perspectives on silicosis management (Leung et al., 2012), silicosis is commonly treated with various therapeutic approaches, including drug therapy with pirfenidone and nintedanib (Bai et al., 2025), lung lavage (Zhang et al., 1997), stem cell therapy (Zhang et al., 2018) and even lung transplantation (Rosengarten et al., 2017). But it still cannot be cured. Currently, the primary clinical drug utilized in China is tetrandrine (Tet), which is a dibenzylisoquinoline alkaloid, used for anti - silicosis (Chan et al., 2021). However, researchers frequently overlook the issue of uneven drug distribution within tissues, which is exacerbated by the transformation of vascular endothelial cells into mesenchymal cells as a consequence of fibrotic diseases. The endothelial-to-mesenchymal transition (EndMT) of blood vessels has been shown to occur in silicosis (Jiang et al., 2021), this may lead to a decrease in the concentration of the drug in the tissue. Therefore, to increase the therapeutic efficacy of Tet, addressing vascular EndMT in silicosis is paramount.

Hydrogen (H_2_), as a gaseous therapeutic agent, can directly penetrate the lungs without requiring systemic absorption, thereby exerting its effects locally. Furthermore, previous studies have shown that hydrogen (H_2_), owing to its potent antioxidant properties, can alleviate oxidative stress (OS) and inhibit endothelial-mesenchymal transition (EndMT) (Ohta, 2012; Yildiz et al., 2025; Li Y. et al., 2024) (Zhang et al., 2024). This mechanistic rationale has supported its application as a therapeutic intervention in chronic obstructive pulmonary disease and related respiratory conditions (Yang et al., 2022; Huang et al., 2025; Liu et al., 2024). We propose that hydrogen could addresses EndMT-mediated barriers in silicosis.

In this study, we aim to establish a mouse model of silicosis in order to systematically investigate how hydrogen supplementation influences the therapeutic efficacy of tetrandrine. Specifically, this work is the first to propose and examine the modulation of endothelial-mesenchymal transition (EndMT) by hydrogen as a mechanistic basis for improved drug delivery and disease outcome in silicosis.

## Materials and methods

### Reagents, drugs and hydrogen devices

SiO_2_ with a diameter of 1–5 μm (Sigma - Aldrich, United States, S5631-100G) were utilized in this study. SiO_2_ was sterilized by high-pressure steamrendered at 120 °C for 2 h. Then resuspend in sterile phosphate-buffered saline (PBS) (Vivacell, China, C3580-0500). Vortex the silica suspension for 1 min before use. N-acetylcysteine (NAC) (MedChemExpress, United States, HY-B0125) was dissolved in 0.9% saline. Tet with a purity greater than 98% (CONBA Co., Ltd., Zhejiang, China, PH2203002A) was resuspended in 1% carboxymethyl cellulose sodium (CMC-Na) (Sigma, United States, C5678-500G) for administration to mice. Hydrogen devices: Hydrogen - oxygen mixed gas (66% hydrogen; 33% oxygen) output at a flow rate of 3 L/min. (Shanghai Asclepius Meditec CO., Ltd., China, model AMS-H-03).

## Animals and treatment

C57BL/6 mice (male, 6–8 weeks old, weighing 20–25 g) were obtained from Beijing Vital River Laboratory Animal Technology Co. Ltd., and were housed in the animal facility of Shandong Academy of Medical Sciences; keep mice at room temperature (25 °C ± 2 °C) with a humidity of 50%–70%, sustain a 12-h light/dark cycle, and there are no restrictions on food and water intake.

The mouse model of silicosis was established by one-time tracheal instillation, as explained previously (Cao et al., 2020). Mice were briefly anesthetized with 2% isoflurane (RWD, R510-22-10), then 50 μL SiO_2_ suspension (0.24 g SiO_2_ in 10 mL sterile PBS) was instilled through their trachea. Mice in the normal control group were given the same volume of PBS in the same way. The mold was successfully made 28 days later. Administration be initiated after successful mold making. We treated all mice according to the procedures approved by the experimental animal ethical committee of the Shandong Academy of Occupational Health and Occupational Medicine (NO.: SDZFY - EC - A- 2023–09). We conduct experiments in accordance with local legislation and institutional requirements.

To detect the content of Tet in the lung tissues of different groups of mice, the mice were divided into three groups include PBS control group, silica group and Tet group. There were ten mice in each group. After successful modeling, they were randomly grouped. PBS control group: after PBS modeling, the mice were gavage - administered 1% CMC-Na for 6 days a week; silica group: after silica modeling, the mice were gavage - administered 1% CMC-Na for 6 days a week; Tet group: after silica modeling, the mice were gavage - administered Tet (20 mg/kg, twice per day) and 1% CMC-Na for 6 days a week. Mice were treated with drugs for 8 weeks.

NAC is a potent antioxidant and anti-inflammatory agent, clinically used to counteract oxidative stress, reduce lung fibrosis, and ameliorate silica-induced inflammation in experimental silicosis models. It works by replenishing intracellular glutathione, scavenging free radicals, and modulating key cytokines such as TNF-α and IL-6 to limit pulmonary fibrosis progression. We divided the mice into PBS control group, silica group and NAC group to verify the therapeutic effect on silicosis mice after improving oxidative stress. There were ten mice in each group. After successful modeling, they were randomly grouped. The NAC group, in which the mice were gavage-administered NAC (3.46 mg/20 g) once a day after silica modeling (Huang et al., 2019). Mice were daily treated with drugs for 8 weeks.

The H_2_ and Tet administration grouping of mice include PBS control group, silica group, H_2_ group, Tet group and H_2_ + Tet group. There were ten mice in each group. After successful modeling, they were randomly grouped. For the H_2_ group, after silica modeling, mice were given the same vehicle (1% CMC-Na) through gavage and made to inhale hydrogen/oxygen mixture for 2 h per day for 6 days a week. For the H_2_ +Tet group, mice were given Tet and inhaled hydrogen/oxygen mixture. The mice were given the indicated agents for 8 weeks.

## Histological examination of the lung tissues

The right upper lung of mice was embedded in paraffin for hematoxylin and eosin (HE) staining and Masson staining. Lung slices were scaned and assessed. The degree of lung injury and pulmonary fibrosis was quantified through scoring.

For immunofluorescence staining, murine lung slices were treated with antibodies against platelet endothelial cell adhesion molecule-1 (CD31) (1:500 dilution, Wuhan Servicebio Technology Co., LTD., China, GB15063-50), CD44 (1:500 dilution, Wuhan Servicebio Technology Co., LTD., China, GB112054), CD68 (1:2000, Wuhan Servicebio Technology Co., LTD., China, GB113109), α-smooth muscle actin (α-SMA) (1:500 dilution, Wuhan Servicebio Technology Co., LTD., China, GB111364), and amyloid precursor protein (APP) (1:500 dilution, Wuhan Servicebio Technology Co., LTD., China, GB11500). Then, the secondary antibody and the cell nucleus were incubated (Servicebio, Wuhan, China, G1012-10 ML). Finally, observed under a fluorescence microscope (Nikon ECLIPSE Ci, Japan, 20×). The degree of staining overlap was quantified using Pearson R value by ImageJ.

### Liquid chromatography-tandem mass spectrometry (LC/MS-MS) analysis

Lung tissue was taken and precipitated proteins at low temperature.

The Thermo UltiMate 3000 high-performance liquid chromatograph (United States) was utilized to achieve chromatographic separation of the target compounds using a Thermo Accucore C18 column (100 mm × 2.1 mm, 2.6 μm) (United States). For mass spectrometry analysis, the AB QTRAP 5500 triple quadrupole mass spectrometer equipped with an ESI ion source was utilized in multi-reaction monitoring (MRM) mode.

All the mass spectrometry data acquisition and quantitative analysis of the target compounds were carried out using the Analyst and multiQuant software respectively.

### Detection of ROS fluorescence levels

Frozen sections were made from lung tissue. For ROS detection, liquid dihydroethidium (DHE) was utilized (Beyotime, Shanghai, China, S0033S). Fluorescence microscopy images were captured using a panoramic digital scanner (TG Tissue FAXS Plus, Austria, 20 ×), and the collected images were subsequently quantified using ImageJ.

### Western blot analysis

Quantified lung tissue homogenate was taken and boiled. Then they were transfer to a polyvinylidene fluoride (PVDF) membrane (Wei et al., 2023). The membranes were incubated with the primary antibodies, namely, Pink1 (1:1000 dilution, Proteintech, United States, 23274-1-AP), LC3 (1:1000 dilution, Cell Signaling Technology, United States, 12741T), sequestosome 1 (SQSTM1/p62) (1:1000 dilution, Cell Signaling Technology, United States, 5114T), Parkin (1:2000 dilution, Proteintech, United States, 14060-1-AP), Kelch-like ECH-associated protein 1 (Keap1) (1:1000 dilution, Proteintech, United States, 60027-1-Ig), nuclear factor E2-related factor 2 (Nrf2) (1:2000 dilution, Proteintech, United States, 16396-1-AP), B- cell lymphoma - 2 (Bcl-2) (1:2000 dilution, Proteintech, United States, 26593-1-AP), Bcl - 2-associated X protein (Bax) (1:5000 dilution, Proteintech, United States, 50599-2-Ig), Cytochrome C (Cyto C) (1:5000, Proteintech, United States, 10993-1-AP), Caspase 3 (1:1000 dilution, Proteintech, United States, 19677-1-AP) and Glyceraldehyde 3-phosphate dehydrogenase (GAPDH) (1:500000 dilution, Proteintech, United States, 60004-1-Ig). Then incubate the secondary antibody. The outcomes were visualized with an enhanced chemiluminescence detection kit (LI-COR Bioscience, United States, 9926–68071) and examined using a gel imaging system (Odyssey Fc Imaging System, LI-COR Biosciences, United States). Use ImageJ for quantification, with GAPDH acting as the internal control.

### Transmission electron microscopy (TEM)

The frozen section, measuring approximately 1 mm^3^, was initially fixed in 2.5% glutaraldehyde (SUPPLIES, United States, 02607-BA) within a phosphate buffer (0.1 M, pH 7.0). Subsequently, the tissue blocks were fixed in 1% osmium tetroxide (Ted Pella Inc., United States, 18456), and after adequate soaking, they were examined using the Hitachi Model H-7800 TEM (Japan).

### Pulmonary function test of mice

Intraperitoneally anesthetized mice with tribromoethanol (0.2 mL/10 g) and subsequently fixed in the supine position on a constant temperature platform maintained at 37 °C. The trachea was incised to facilitate tracheal intubation and was then connected to a small animal pulmonary function instrument (model BUXCO PFT, United States) for the collection of respiratory resistance data.

### Computed tomography (CT) imaging of the lung

To fully expose the lung scanning area, we anesthetized mice with 2% isoflurane and positioned in a prone position on the plate, with their extremities extended. A scan was performed from the neck to the diaphragm using the Micro-PRT/CT system (IRIS PET/CT, Inviscan, Strasbourg, France). The CT images were quantified using the HU value.

### Detection of hydroxyproline (HYP) level in the lung tissue samples

Hydroxyproline is a major component of collagen and its quantification serves as a widely accepted biochemical marker for evaluating the extent of pulmonary fibrosis. Measuring HYP provides an objective and quantitative assessment of collagen deposition and disease severity, making it the “gold standard” for lung fibrosis studies and therapeutic intervention assessment. After the sample is hydrolyzed with alkaline reagents, free HYP is generated. Under the action of an oxidizing agent, the oxidation product produced by it reacts with the color-developing agent to form a purple-red color. This substance has an absorption peak at 565 nm. Within a certain range, its absorbance is linearly related to the concentration. Hydroxyproline level was measured with a HYP measurement kit (Nanjing Jian Cheng Institute, China, A030-2-1) rigorously based on the instruction book from manufacture.

### Determination of OS markers in the lung tissue samples

The OS markers superoxide dismutase (SOD) (Solarbio, Beijing, China, BC0175) and malondialdehyde (MDA) (Solarbio, Beijing, China, BC0025) were detected using biochemical kits. Measurements were conducted following the manufacturer’s instructions.

### TdT-mediated dUTP nick - end labeling (TUNEL) assay

Lung tissue samples were prepared into paraffin sections and utilized to detect apoptosis through the TUNEL staining kit (Wuhan Servicebio Technology Co., Ltd., China, G1504-50T). Subsequently observed under a fluorescent microscope (Nikon ECLIPSE Ci, Japan, 20×). Quantification was performed using ImageJ software.

### Single-cell suspension preparation

Lung tissue was freshly collected for single-cell dissociation. Dissociate and digest cells, and conduct cell counting and viability assessment.

### Single-cell RNA-seq

The 10× Genomics Chromium Next GEM Single Cell 3ʹ Kit v3.1 (1000268, 10x Genomics, Inc., United States) was utilized for scRNA-seq following the instructions in the user guide (CG000315). Then we performed Visualization of antioxidant gene expression using the Seurat R package and CellChat analysis using the CellChat (Jin et al., 2021) R package (version 1.1.3.). Quantitative analysis was performed using cellranger processing. OE Biotech Co., Ltd. (Shanghai, China) conducted the sequencing and bioinformatics analysis.

### Bioinformatic analysis

In this study, the Search Tool for the Retrieval of Interacting Genes (STRING: version 12.0, https://cn.string-db.org/) online database was used to predict the CD74 interaction network.

Gene Ontology (GO) enrichment analysis was performed using the Database for Annotation, Visualization and Integrated Discovery (DAVID) bioinformatics resources (version 6.8) (https://davidbioinformatics.nih.gov/tools.jsp). Initially, the official symbols of the target genes, CD74 (Gene ID: 16176) and CD44 (Gene ID: 15481), were entered into the DAVID analysis system. The analysis was restricted to *Mus musculus*, with the entire genome annotation utilized as the background dataset. The system automatically aligns annotation information from databases such as Kyoto Encyclopedia of Genes and Genomes (KEGG) and UniProt, generating a comprehensive analysis report that includes GO entries, enrichment factors, P-values, and False Discovery Rate (FDR) values.

### Statistical analysis

Results are presented as the mean ± standard deviation (SD). A two-tailed Student’s t-test was used to compare two groups and a one-way ANOVA and Bonferroni Post-hoc Analysis were used for comparing multiple groups. GraphPad Prism 9.0 software and SPSS 27.0 was used to perform all statistical analyses. A p-value <0.05 was used to determine statistical significance for group comparisons.

## Results

### Tet concentration decreased in lung tissue and oxidative stress excessive activated in silicosis

In this subsection, we found that after the establishment of the silicosis model, the concentration of Tet in lung tissue decreased from 94.8 ± 10.4 ng/mg to 54 ± 6.2 ng/mg.

**FIGURE 1:**
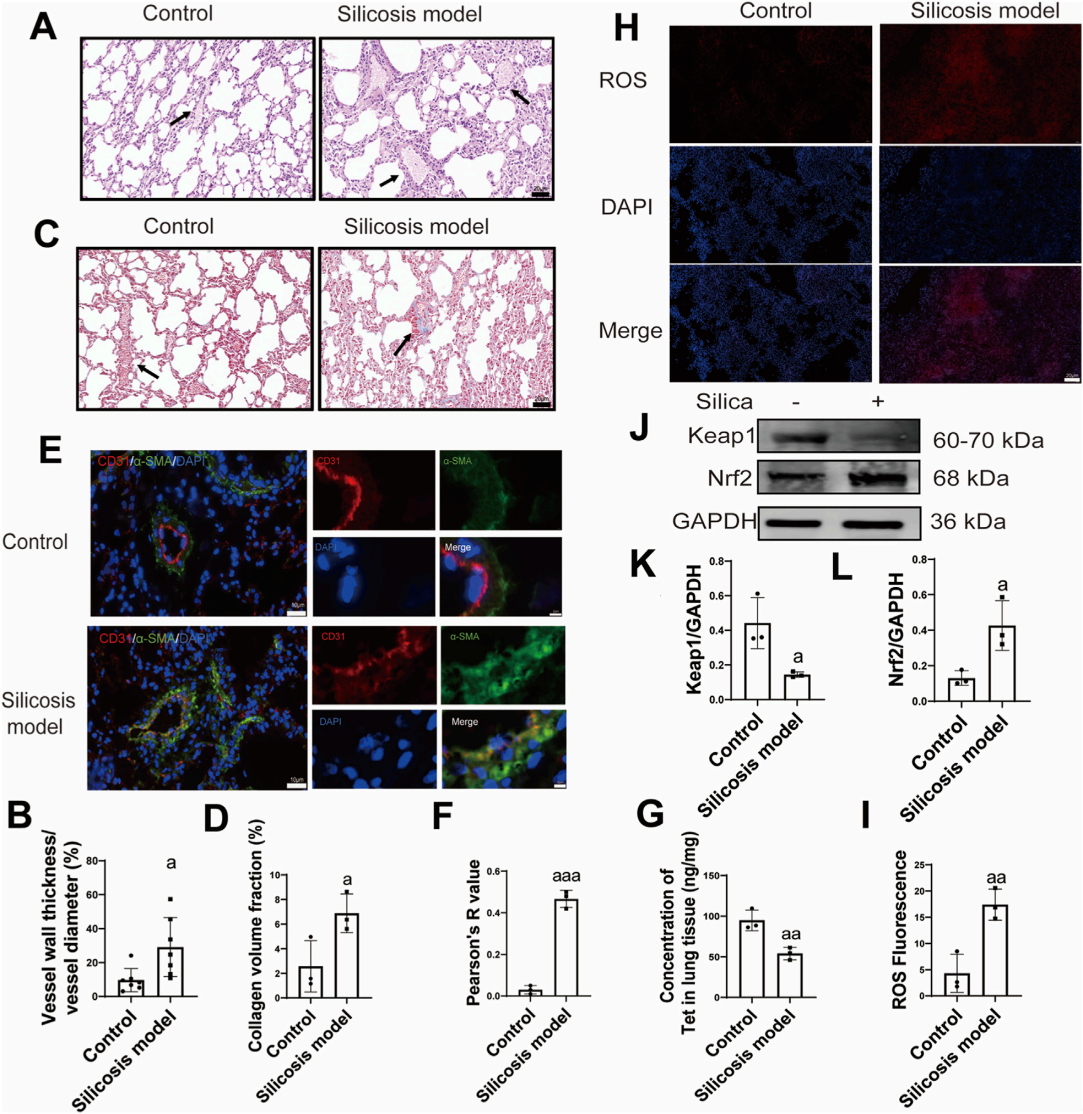
Tetrandrine concentration decreased in lung tissue and oxidative stress excessive activated in silicosis. **(A,B)**, HE staining and quantitative analysis of the degree of vascular stenosis of each group of mice (n = 3), scale bar, 20 μm; **(C,D)**, Masson staining and collagen volume fraction of each group of mice (n = 3), scale bar, 20 μm; **(E,F)**, Immunofluorescence and semi-quantitative of the expression of the endothelial to mesenchymal transition (EndMT)-related proteins platelet endothelial cell adhesion molecule-1 (CD31) and alfa-smooth muscle actin (α–SMA) in the lung tissues of different mouse groups (n = 3). Scale bar, 10 μm and 2 μm, Pearson’s R is the correlation coefficient—a statistical measure that indicates both the strength and direction of a linear relationship between two continuous variables, ranging from −1 (strong negative) to +1 (strong positive); **(G)**, The concentration of tetrandrine in lung tissue was detected by LC/MS-MS (n = 3); **(H,I)**, Changes of ROS levels in the lung tissues (n = 3). Scale bar, 20 μm, ROS: reactive oxygen species, DAPI: 4′,6-Diamidino-2-phenylindole dihydrochloride, Merge: ROS combines with the cell nucleus; **(J)**, Western blotting of Keap1 and Nrf2 expression in the lung tissues (n = 3); **(K,L)**, Semi-quantitative of the expression of Keap1 and Nrf2 in the lung tissues (n = 3). ^a^P < 0.05; ^aa^P < 0.01 vs. the control group. Data are expressed as mean ± SD.

The thickness of the vascular wall was detected by HE staining. The HE staining results demonstrated that the vascular walls in the silicosis mouse model were thickened (p = 0.033, 95%CI = −29.24 to −2.06) (Figures 1A,B). Collagen deposits on the vascular wall were detected by Masson staining. Masson staining indicated an increase in collagen deposition around the vascular walls of silicosis mice (p = 0.046, 95%CI = −8.51 to −0.11) (Figures 1C,D). This vascular wall stenosis may lead to reduced blood flow, subsequently decreasing the drug concentration in lung tissue. Double immunofluorescence staining were conducted to evaluate EndMT. The analysis revealed that the proportion of CD31^+^/α-SMA^+^ double-positive cells in the pulmonary vessels of the silicosis group was increased (p < 0.001, 95%CI = −0.51 to −0.36) (Figures 1E,F). This may lead to the thicken of the blood vessel wall and the drug concentration in lung tissue decreased. Figure 1G shows that the concentration of Tet in silicosis mice is lower than that observed in normal mice (p = 0.009, 95%CI = 16.96–64.64).

OS usually causes EndMT. We examined the degree of OS in silicosis model mice. DHE fluorescence probes indicated that the fluorescence intensity of ROS in the alveolar region and silica nodules region of the silicosis group was markedly increased (p = 0.009, 95%CI = −20.65 to −5.55) (Figures 1H,I), suggesting a compensatory activation of both OS and the antioxidant system. Furthermore, Western blot analysis indicated a downregulation of the OS-related protein Keap1 (p = 0.025, 95%CI = 0.06–0.53) and an activation of Nrf2 (p = 0.025, 95%CI = −0.53 to 0.06) (Figures 1J–L).

### Excessive activation of oxidative stress leads to inhibition of mitochondrial autophagy and excessive apoptosis

In this subsection, we found that after the activation of oxidative stress, the expression level of the apoptosis marker protein caspase3/GAPDH increased from 0.18 ± 0.01 to 0.34 ± 0.05.

**FIGURE 2:**
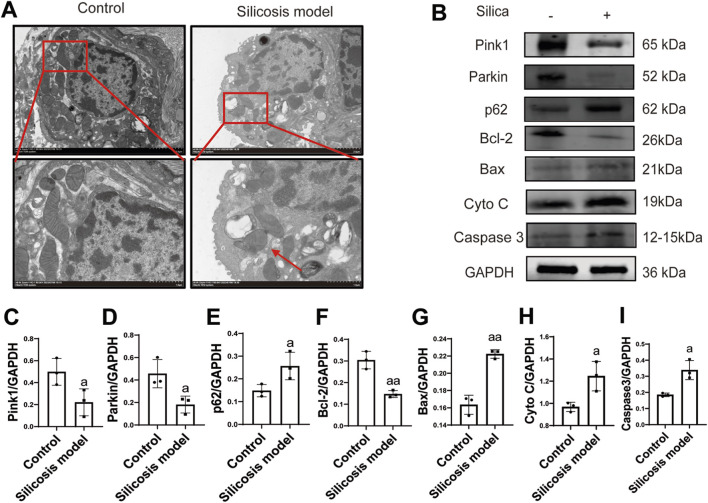
Excessive activation of oxidative stress leads to inhibition of mitochondrial autophagy and excessive apoptosis. **(A)**, Mitochondrial injury observed under transmission electron microscopy (TEM) (n = 3). Scale bar, 2.0 (×4.0k) and 1.0 (×8.0k) μm; **(B)**, Western blotting of Pink1, Parkin, p62, Bcl2, Bax, Cyto C and Caspase3 expression in the lung tissues (n = 3); **(C–I)**, Semi-quantitative of the expression of Pink1, Parkin, p62, Bcl2, Bax, Cyto C and Caspase3 in the lung tissues (n = 3). ^a^P < 0.05; ^aa^P < 0.01 vs. the control group. Data are expressed as mean ± SD.

Regarding mitochondrial quality control regulation, TEM revealed significant pathological changes in the mitochondria of lung tissue cells from the silicosis group: the structure of the mitochondrial cristae was blurred or absent, the integrity of the mitochondrial membrane was compromised, and the mitochondria number was reducted (Figure 2A). Western blot analysis indicated that the mitophagy marker Pink1 (p = 0.049, 95%CI = 0.00–0.55) and Parkin (p = 0.031, 95%CI = 0.04–0.51) were decreased in the silicosis group, while p62 (p = 0.048, 95%CI = −0.21 to 0.00) was upregulated (Figures 2B–E), suggesting that autophagic flow was inhibited. These findings imply that the impaired mitochondrial autophagy induced by SiO_2_ may be closely linked to the disruption of mitochondrial quality control.

Western blot analysis detected apoptosis-related proteins, the results indicated that the anti-apoptotic protein Bcl-2 was inhibited in silicosis (p = 0.005, 95%CI = 0.20–0.31), while the pro-apoptotic protein was activated (p = 0.016, 95%CI = −0.08 to −0.02). Meanwhile, both the mitochondrial-mediated apoptotic proteins Cyto C (p = 0.026, 95%CI = −5.0 to −0.05) and caspase3 (p = 0.043, 95%CI = −3.0 to −0.01) were activated (Figures 2B,F–I).

### Inhibiting oxidative stress can improve the concentration of tet in lung tissue in mice with silicosis

In this subsection, we found that after inhibiting oxidative stress, the concentration of Tet in the lung tissue of silicosis mice increased from 41.9.8 ± 6.4 ng/mg to 71.8 ± 12.4 ng/mg.

**FIGURE 3:**
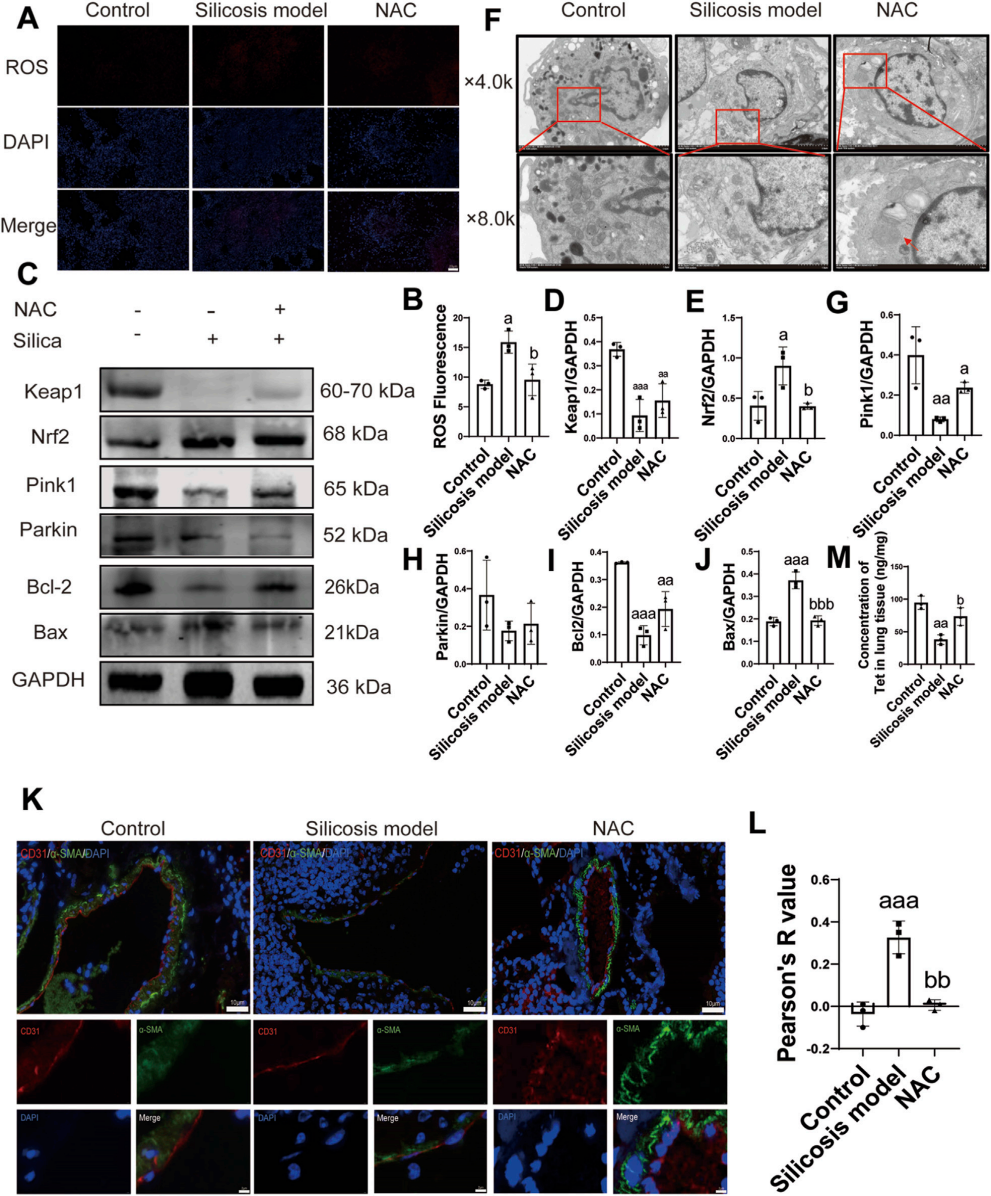
Inhibiting oxidative stress can improve the concentration of tetrandrine in lung tissue in mice with silicosis. **(A,B)**, Changes in the ROS levels in the lung tissues (n = 3). Scale bar, 20 μm, ROS: reactive oxygen species, DAPI: 4′,6-Diamidino-2-phenylindole dihydrochloride, Merge: ROS combines with the cell nucleus; **(C)**, Western blotting of Keap1, Nrf2, Pink1, Parkin, Bcl-2 and Bax expression in the lung tissues (n = 3); **(D,E)**, Semi-quantitative of the expression of Keap1 and Nrf2 in the lung tissues of different mouse groups (n = 3); **(F)**, Mitochondrial injury observed under transmission electron microscopy (TEM) (n = 3). Scale bar, 2.0 (×4.0k) and 1.0 (×8.0k) μm; **(G–J)**, Semi-quantitative of the expression of Pink1, Parkin, Bcl-2 and Bax in the lung tissues of different mouse groups (n = 3); **(K,L)**, Immunofluorescence and semi-quantitative of the expression of the endothelial to mesenchymal transition (EndMT)-related proteins platelet endothelial cell adhesion molecule-1 (CD31) and alfa-smooth muscle actin (α–SMA) in the lung tissues of different mouse groups (n = 3). Scale bar, 10 μm and 2 μm; **(M)**, The concentration of tetrandrine in lung tissue was detected by LC/MS-MS (n = 3 biologically independent experiments). ^a^P < 0.05; ^aa^P < 0.01; ^aaa^P < 0.001 vs. the control group. ^b^P < 0.05; ^bb^P < 0.01 vs. the model group. Data are expressed as mean ± SD.

We administered NAC to mice and assessed the concentration of Tet in lung tissue. First, the DHE fluorescence intensity of ROS in the silicosis group was elevated compared to the control group (p = 0.012, 95%CI = −12.26 to −1.94), whereas ROS production significantly decreased following NAC intervention (p = 0.02, 95%CI = 1.19–11.51), suggesting that NAC effectively mitigated oxidative damage induced by silicosis (Figures 3A,B). Western blot analysis indicated that the Keap1 expression in silicosis mice was markedly reduced, while the expression of Nrf2 (p = 0.038, 95%CI = −0.96 to −0.03) was increased. However, after NAC administration, the expression levels of these proteins returned to near-normal ranges (p = 0.036, 95%CI = 0.039–0.96) (Figures 3C–E), indicating that NAC effectively inhibits the excessive activation of the OS pathway.

Secondly, the situation of mitochondrial autophagy was explored. Electron microscopy observations revealed that mitochondria in the silicosis group exhibited pronounced swelling and rupture of cristae structures. In contrast, compared to the silicosis group, the number of mitochondrial autophagic vesicles in the NAC group increased, and typical autophagosome structures enveloped in double membranes were observed (Figure 3F). The assessment of mitochondrial autophagy indicated that NAC intervention significantly upregulated Pink1 and Parkin expression involved in mitophagy (Figures 3C,G,H), demonstrating that the inhibition of OS can enhance Pink1-mediated mitochondrial autophagy.

Then, the expression of the apoptosis-related proteins Bcl-2 and Bax was assessed, and both were found to be restored after NAC treatment (Figures 3C,I,J).

We further evaluated the EndMT process through double immunofluorescence staining using α-SMA and CD31. The results indicated that the positive rate of α-SMA in vascular endothelial cells from the silicosis group was higher than control group (p < 0.001, 95%CI = −0.52 to −0.21). Notably, after NAC intervention, the proportion of co-expressing cells decreased (p = 0.001, 95%CI = 0.16–0.47). This finding suggests that the inhibition of OS can effectively impede the EndMT level (Figures 3K,L).

Finally, we verified that the concentration of tetrandrine in lung tissue increased after administration of NAC (p = 0.019, 95%CI = 7.05–64.14) (Figure 3M).

### H_2_ can improve the tet concentration in lung tissue and enhance its therapeutic effect

In this subsection, we found that after adding H_2_, the concentration of Tet in the lung tissue of silicosis mice increased from 38 ± 6.1 ng/mg to 73.4 ± 11 ng/mg.

**FIGURE 4:**
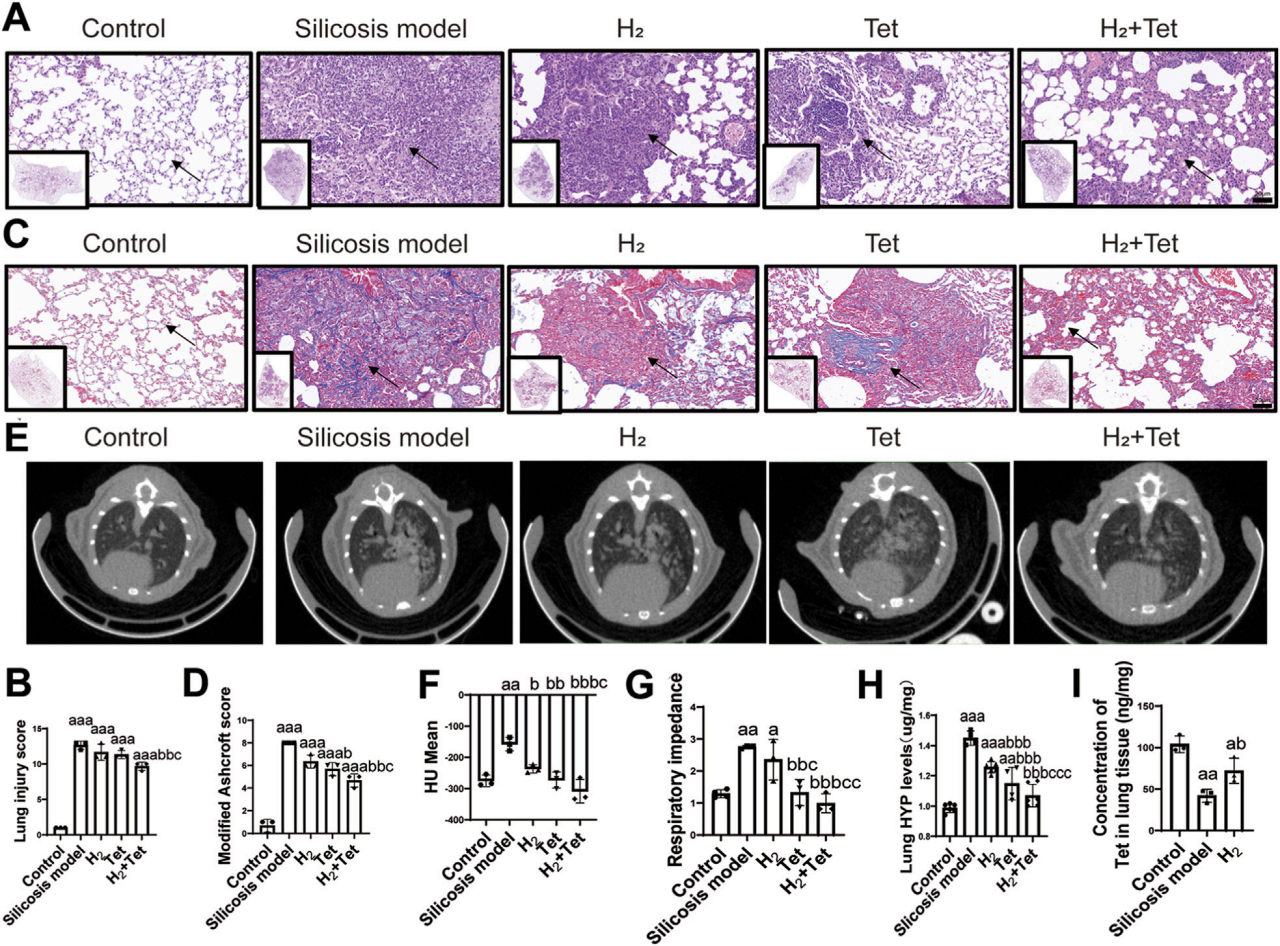
H_2_ can improve the Tet concentration in lung tissue and enhance its therapeutic effect. **(A,B)**, HE staining and lung injury score of each group of mice (n = 3), scale bar, 20 μm; **(C,D)**, Masson staining and Modified Ashcroft scoring (A way to score the degree of silicosis fibrosis) of each group of mice (n = 3), scale bar, 20 μm; **(E,F)**, Computed tomography in each group of mice (n = 3), HU: The Hounsfield units (HU) of lung CT values are quantitative indicators for measuring tissue density, and are used to distinguish different lung structures and lesions, the HU of mice with severe silicosis ranges from −200 to +100; **(G)**, Respiratory resistance in pulmonary function tests in each group of mice (n = 3); **(H)**, Hydroxyproline levels in the lung tissues of different groups of mice (n = 4–6); **(I)**, The concentration of tetrandrine in lung tissue was detected by LC/MS-MS (n = 3). ^a^P < 0.05; ^aa^P < 0.01; ^aaa^P < 0.001, compared with control group. ^b^P < 0.05; ^bb^P < 0.05; ^bbb^P < 0.001, compared with model group. ^c^P < 0.05, compared with H2 group. Data are expressed as mean ± SD.

HE staining indicated that in the silicosis model group, there exits typical destruction of alveolar structures, infiltration of inflammatory cells, and interstitial thickening in lung tissue. In the H_2_ and Tet treatment groups administered individually, the degree of inflammatory infiltration decreased. However, in the combined treatment group, the density of inflammatory cells decreased further, and the integrity of the alveolar structure was significantly restored (p = 0.003, 95%CI = 1.0–5.0) (Figures 4A,B). Masson staining revelaed that the area of collagen fiber deposition in the H_2_ + Tet group was reduced compared to the silicosis group (p < 0.001, 95%CI = 1.82–4.84), and this reduction was greater than that observed in the H_2_ or Tet monotherapy groups (Figures 4C,D). Lung CT and respiratory resistance analyses of the mice demonstrated that the model group exhibited typical diffuse high-density nodules and functional decline. While improvements were noted in the monotherapy groups, the effects were not as pronounced as those observed in the combination therapy group (Figures 4E–G). This finding aligns with the trend of collagen content reduction indicated by lung HYP levels, the “gold standard” for lung fibrosis studies and therapeutic intervention assessment (Figure 4H), confirming that the combined treatment can synergistically inhibit the progression of pulmonary fibrosis. Further exploration of the reasons for the enhanced efficacy observed with combined medication revealed that, in the combined medication group, there was a higher concentration of the drug in lung tissue (p = 0.042, 95%CI = −60.44 to 0.78). This increased concentration enables a more effective therapeutic effect (Figure 4I).

To further investigate the mechanism by which H_2_ enhances Tet concentration in lung tissue and to determine whether its effects are mediated through the modulation of OS, we examined the relevant pathways associated with OS. DHE fluorescence probe revealed that the ROS levels in the combined treatment group were lower than silicosis group and significantly more effective than in the monotherapy group. Notably, the inhibitory effect of H_2_ surpassed that of Tet (p < 0.001, 95%CI = 27.27–58.43) (Supplementary Figures S1A,B). These findings were corroborated by the assessment of biomarkers associated with OS. The combined treatment restored SOD activity to normal levels and reduced MDA content to baseline levels, with H_2_ alone demonstrating a superior therapeutic effect compared to Tet alone (Supplementary Figures S1C,D). Additionally, the Keap1/Nrf2 signaling pathway expression levels were analyzed via Western blot. The results were consistent with the OS markers (Supplementary Figures S1E–G).

Further investigations into mitochondrial functions directly associated with OS. Western blot analysis showed a stronger activation of Pink1 in H_2_ group compared to both the silicosis group and the Tet group. Notably, in the combined treatment group, the activation level of the Pink1/Parkin pathway peaked, suggesting a restoration of autophagic flux (Supplementary Figures S1E,H–K). Additionally, TEM demonstrated a significant increase in the number of mitochondrial autophagic vesicles across all treatment groups, with the morphological integrity of autophagosomes improving in accordance with the intensity of the treatment (Supplementary Figure S1L). It is indicated that H_2_ alone or in combination with Tet can improve mitochondrial autophagy through improve OS.

Accoding to TUNEL staining, the apoptosis cells in lung tissue in silicosis group was elevated (p = 0.003, 95%CI = −51.6 to 10.95), whereas in both the H_2_ monotherapy group and the Tet monotherapy group exhibited a decrease. Notably, the apoptosis rate in the H_2_ group was marginally superior to that in the Tet group. The combined intervention group demonstrated the lowest apoptosis rate, significantly outperforming each monotherapy group (Supplementary Figures S2A,B).

Furthermore, the co-localization coefficients of α-SMA and CD31 in combined treatment group also decreased in comparison to the silicosis group (p < 0.001, 95%CI = 0.17–0.42) (Supplementary Figures S2C,D). Additionally, the H_2_ and combination treatment groups exhibited a reduction in thickening of the blood vessel wall (p < 0.001, 95%CI = 5.65–21.39) and collagen deposition around the vascular wall (p < 0.001, 95%CI = 4.0–13.73) by ameliorating EndMT (Supplementary Figures S2E–H). This suggests that the combination of H_2_ enhances the concentration of Tet in lung tissue may by alleviating vascular stenosis.

### H_2_ combined with tet play a role in endothelial cells and macrophages

**FIGURE 5:**
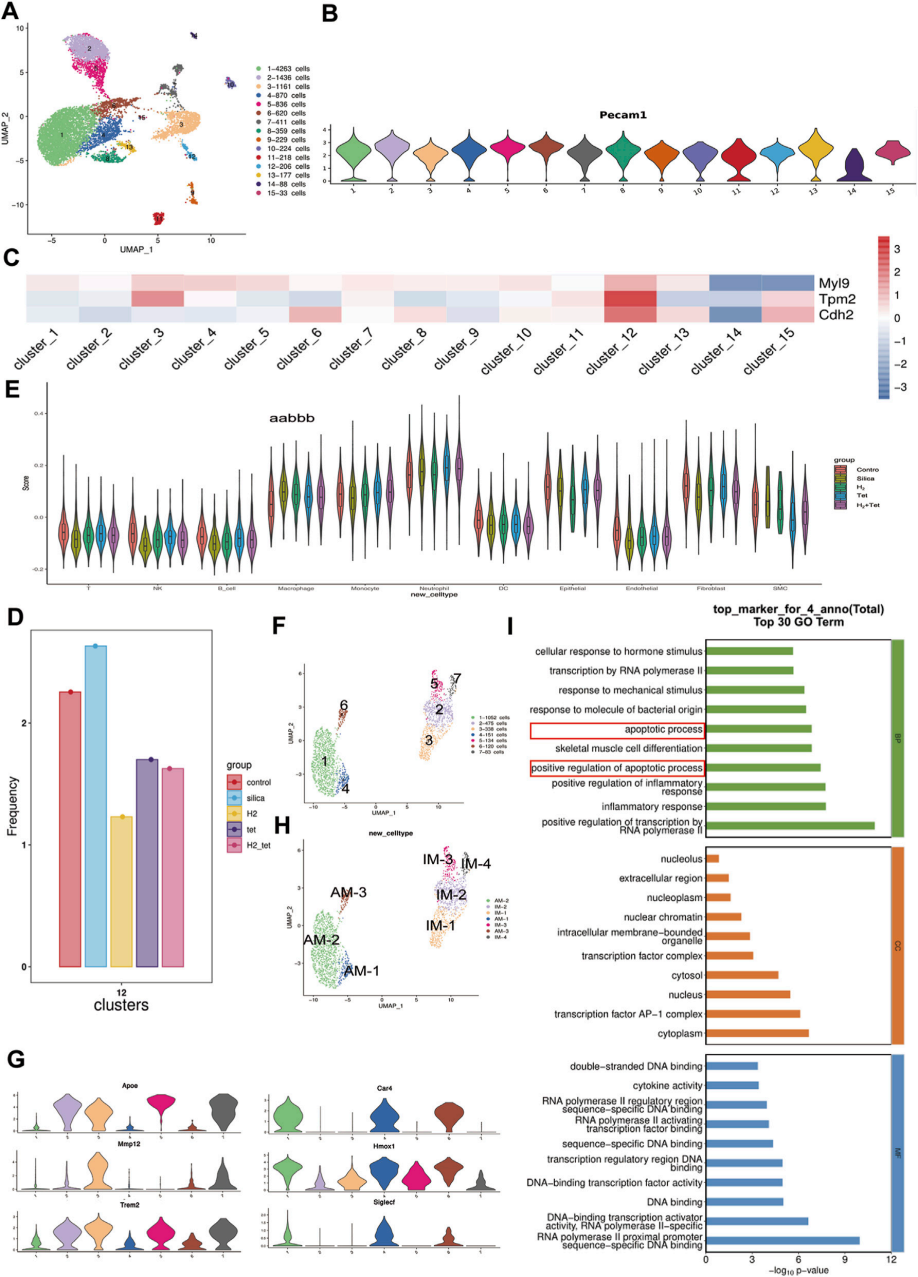
H_2_ combined with tetrandrine play a role in endothelial cells and macrophages. **(A)**, UMAP embedded visualization of different EC subpopulations (n = 11,131 cells); **(B)**, Violin plots showed expression of vascular EC marker gene; **(C)**, Heatmap showing differentially expressed EndMT marker genes in each EC subpopulation; **(D)**, Cell frequencies of each administration group in Cluster 12 of endothelial cells; **(E)**, Violin plot showed the feature of antioxidation activity in the different groups of mouse lung clusters; **(F)**, UMAP embedded visualization of different macrophage subpopulations (n = 2,353 cells); **(G)**, Violin plots showing expression of vascular AM and IM marker gene; **(H)**, UMAP embedded visualization of AM and IM (n = 2,353 cells); **(I)**, GO analysis of top marker for AM-1 (clusters 4). ^aa^P < 0.01, compared with control group. ^bbb^P < 0.001, compared with model group.

We isolated and sequenced 9600 ∼ 17132 cells from whole lung cell suspensions of five male mice, including one control mice, one silicosis mice, one silica and H_2_ - exposed mice, one silica and Tet - exposed mice and one silica, H_2_ and Tet - exposed mice. After quality control, the number of cells obtained was 8300–13158. Of these, 13158 cells originated from control lungs, 8300 cells from silica - infected lungs, 9702 cells from H_2_ group, 10840 cells from Tet group and 8500 cells from H_2_ + Tet group. We conducted dimension reduction with t - SNE subspace alignment, followed by an unsupervised clustering assay. We classified 11 distinct cell clusters, each cluster consisted of as few as 99 cells to 6712 cells (Supplementary Figure S3A). The representative markers identified 11 clusters, including T cells (*Cd3e*, *Cd3d*, *Cd3g*), NK cells (*Nkg7*, *Gzmb*), B cells (*Cd19*, *Cd79a*, *Cd79b*), macrophages (*C1qa*, *C1qb*, *C1qc*, *Mrc1*), monocytes (*S100a4*, *Plac8*, *Ccr2*), neutrophils (*S100a8*, *S100a9*), DC (*Flt3*, *Siglech*), epithelial cells (*Epcam*, *Cdh1*, *Krt18*, *Krt8*), endothelial cells (*Pecam1*, *Cdh5*, *Vwf*), fibroblast cells (*Col1a1*, *Col1a2*, *Col3a1*) and SMC (*Acta2, Tagln*, *Myh11*) (Supplementary Figure S3B). We conducted dimension reduction with t - SNE subspace alignment, followed by an unsupervised clustering assay. We identified endothelial cells (*Pecam1*, *Cdh5*, *Vwf*), divide into 15 clusters (Figure 5A). Then we found vascular endothelial cells (*Pecam1* ^+^), all 15 clusters belong to (Figure 5B). Among them, the subgroups with endothelial-mesenchymal transition (*CDH2* ^+^, *MYL*9^+^ and *TPM2* ^+^) are cluster 12 (Figure 5C). According to cell frequcy, both the hydrogen and combination therapy groups showed a decrease compared to the model group (Figure 5D).

Single cell sequencing analysis indicated that H_2_ and Tet significantly regulated the antioxidant stress activity in macrophages. The expression levels of antioxidant-related genes in macrophages were significantly downregulated in the H_2_ monotherapy group, the Tet monotherapy group, and the combination therapy group (H_2_+Tet) compared to the silicosis group, indicating a synergistic effect of the two agents by inhibiting the OS pathway in macrophages (Figure 5E). We identified macrophages (*C1qa*, *C1qb*, *C1qc*, *Mrc1*), divide into 6 clusters (Figure 5F). In the lungs, macrophages are categorized into pulmonary interstitial macrophages (IM) and alveolar macrophages (AM). We classified macrophages into AM (*Car4* ^+^, *Homx1* ^+^ and *Siglecf* ^+^) and IM (*Apoe* ^+^, *Mmp12* ^+^ and *Trem2* ^+^) according to Cell Marker 2.0 (Figures 5G,H). The primary function of AM-1 (cluster 4) is associated with apoptosis process and positive regulation of apoptosis process (Figure 5I). So we focused on AM-1.

### H_2_ combined with tetrandrine can improve the APP/CD74 interaction

In this subsection, we found that after adding H_2_, the prob value for communication between AM-1 and endothelial cells decreased from 0.63 to no communication effect.

**FIGURE 6:**
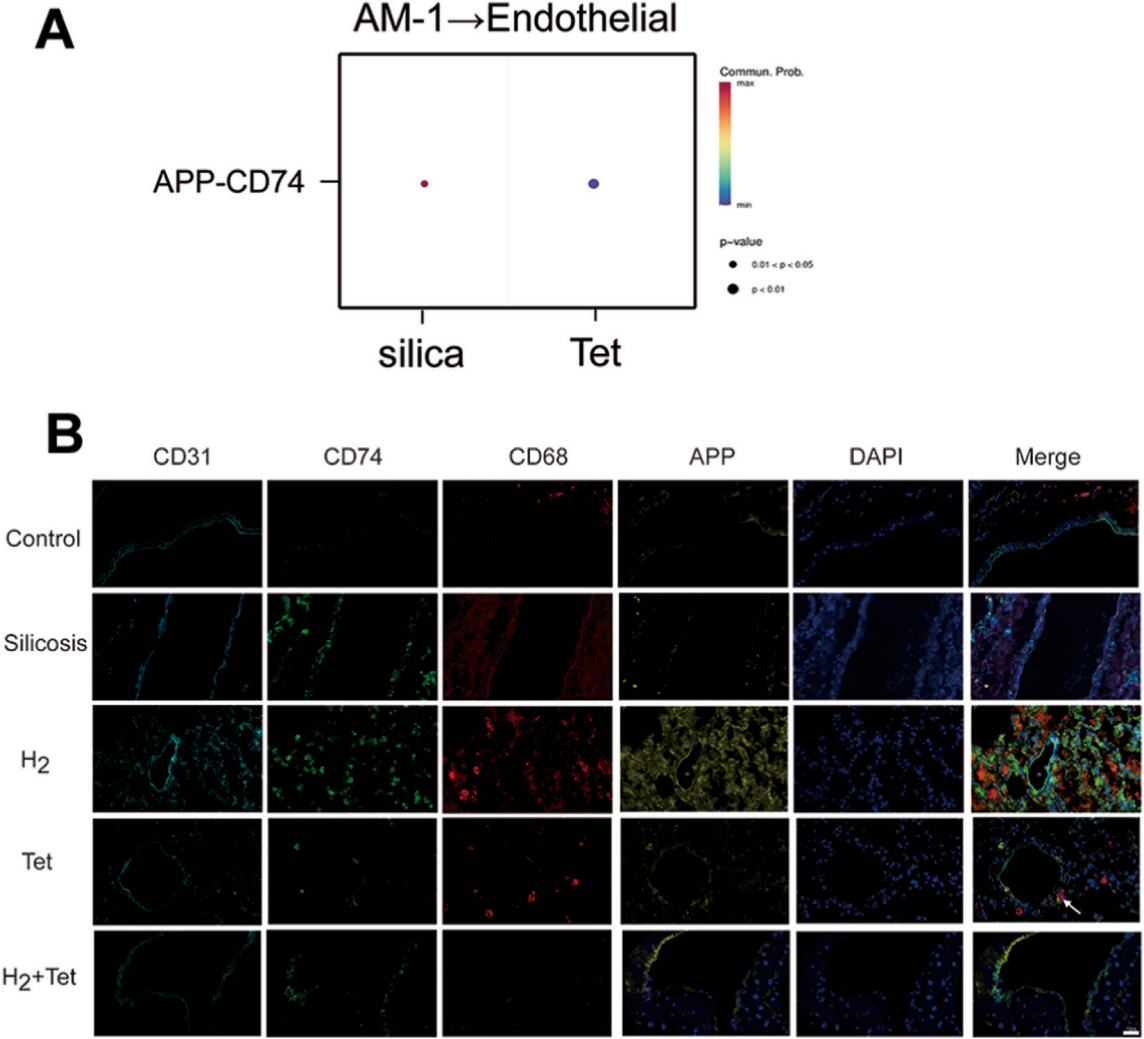
H_2_ combined with tetrandrine can improve the APP/CD74 interaction. **(A)**, CellChat algorithm of alveolar macrophages to Endothelial cellC; **(B)**, Immunofluorescence of the expression of CD31, CD74, CD68 and APP in the lung tissues of different mouse groups (n = 3). Scale bar, 20 μm and 10 μm.

We employed the CellChat algorithm to investigate the communication network between AM-1 subsets and endothelial cells. The results indicated that the APP/CD74 ligand-receptor pair exhibited a significantly activated state in the silicosis model. Notably, the signal intensification of APP/CD74 was absent in both the H_2_ monotherapy group and the H_2_ + Tet group, while the inhibitory effect of the combination therapy was significantly superior to that of the Tet monotherapy group (Figure 6A). These findings suggest that H_2_ may enhance the inhibitory effect of Tet on macrophage apoptosis-related communication by selectively targeting and regulating the APP/CD74 axis. Then we verified it through immunofluorescence. The AM marker CD68 and its secreted APP were co-stained with the vascular endothelial marker CD31 and CD74 expressed in vascular endothelial cells. It was found that CD68 and APP interacted with CD74 on CD31 in the silicosis group and the Tet group, but not in the normal group, the H_2_ group and the H_2_ + Tet group (Figure 6B).

### H_2_ combined with tetrandrine has the effect of inhibiting CD74/CD44-mediated endothelial mesenchymal transition

In this subsection, we found that after adding H_2_, the Pearson R value of EndMT marker proteins CD44 and CD31 decreased from 0.38 ± 0.05 to −0.06 ± 0.05.

**FIGURE 7:**
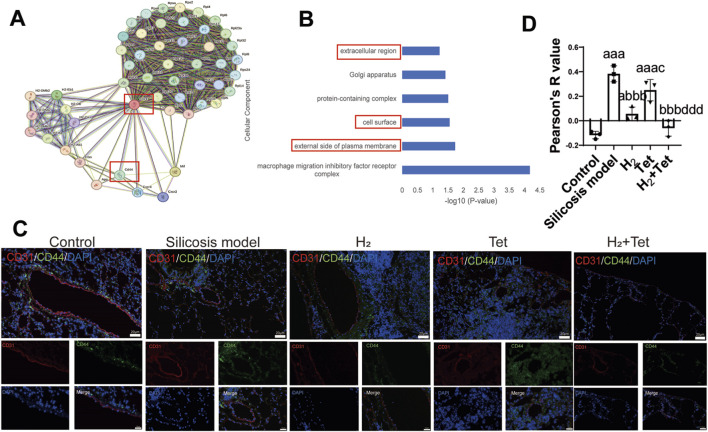
H_2_ combined with tetrandrine has the effect of inhibiting CD74/CD44-mediated endothelial mesenchymal transition. **(A)**, The protein interaction network of CD74 was plotted through the string database.; **(B)**, GO enrichment analysis was conducted on CD74 and CD44, and the results were biological processes related to endothelial mesenchymal transition; **(C,D)**, Immunofluorescence and semi-quantitative of the expression of CD44 and platelet endothelial cell adhesion molecule-1 (CD31) in the lung tissues (n = 3). Scale bar, 20 μm and 10 μm ^a^P < 0.05; ^aaa^P < 0.001, compared with control group. ^bbb^P < 0.001, compared with model group. ^c^P < 0.05, compared with H_2_ group. ^ddd^P < 0.001, compared with Tet group. Data are expressed as mean ± SD.

Protein-protein interaction network of CD74 indicated a strong co-expression between CD74 and CD44, a key marker of EndMT, with an interaction index of 0.998 (confidence threshold >0.9). This suggests that CD74 and CD44 may regulate EndMT by forming complexes or sharing signaling pathways (Figure 7A). Additionally, a GO enrichment analysis of CD74 and CD44 revealed that the enriched pathways were highly consistent with the extracellular matrix and the cell surface interactions during EndMT (Figure 7B). According to these results, we speculate that H_2_ + Tet may enhance the EndMT process by targeting the CD74/CD44-mediated signaling axis. Immunofluorescence of CD44 vascular endothelial cells demonstrated that, the fluorescence intensity of CD44 in the H_2_ + Tet treatment group was significantly reduced compared to silicosis group (p < 0.001, 95%CI = 0.26–0.63) (Figures 7C,D).

## Discussion

We used many kinds of methods to investigate possible causes of low concentration of tetrandrine in lung tissue in silicosis in our study. Using single-cell sequence, further investigate the possible mechanism by which H_2_ enhances Tet’s efficacy. In conclusion, our research provides strong evidence that H2 adjuvant therapy for silicosis enhances the efficacy of Tet and offers valuable insights into the potential mechanisms underlying the treatment of this disease.

Silicosis has imposed a significant economic burden on society due to the absence of effective treatments (Akgun and Ergan, 2018). Many scholars have revealed the relationship between OS and pulmonary fibrosis (Zhao et al., 2020; Tian et al., 2023). For instance, OS is crucial for the progression of silicosis by damaging lung tissue and promoting inflammatory responses (Langley et al., 2011; Wang et al., 2022). Currently, no study has demonstrated how OS impacts the efficacy of drug treatments by influencing the vascular wall thickness and EndMT of silicosis. Furthermore, the influence of OS in other cell types on the EndMT in vascular endothelial cells has been even less explored. Our work revealed the significant interrelationships among OS, mitochondrial autophagy, apoptosis and EndMT in the Tet poor therapeutic effect condition of silicosis.

Although H_2_ has been applied in clinical practice (Yang et al., 2022; Huang et al., 2025; Liu et al., 2024) and experiments have shown that its combination with Tet can improve silicosis (Li J. et al., 2024), the specific mechanism has not yet been clarified. Hydrogen has been applied in various diseases, and its most prominent effect is antioxidation. Ohsawa indicated that hydrogen can reduce cytotoxic oxygen radicals (Ohsawa et al., 2007). However, the most significant pathological change of silicosis is the excessive activation of OS. Therefore, we consider applying H_2_ to the antioxidant effect of silicosis. Given that ROS are predominantly generated in mitochondria, excessive OS activation can lead to mitochondrial damage and mitophagy (Szeto, 2006; Ikawa and Yoneda, 2009; Andreyev et al., 2005), which facilitates the release of Cyto C from mitochondria into the cytoplasm, thereby triggering apoptosis (Liang et al., 2013; Yu et al., 2008). We explored the effects of H_2_ combined with Tet on OS, mitochondrial autophagy and apoptosis.

Then, we explored the relationship between OS and EndMT by single-cell RNA sequencing analysis. Single-cell RNA sequencing analysis has been applied in silicosis (Song et al., 2022), but the interaction between cells has not yet been discovered. The observed improvement in EndMT through the inhibition of OS may be attributed to intercellular communication between alveolar macrophages and endothelial cells. Notably, the ligand pair APP/CD74 was found to be absent in both hydrogen and the combined treatment. Previous studies have shown that APP is abnormally expressed in silicosis (Liu et al., 2016; Liu et al., 2019), which is consistent with our results. Immunofluorescence analysis revealed that apoptosis-related macrophage release of APP, which subsequently interacts with CD74 in endothelial cells. The robust interaction between CD74 and CD44 further modulates EndMT. EndMT is when endothelial cells lose their phenotypic characteristics and acquire mesenchymal or stem cell-like properties, and CD44, as a cell adhesion molecule, can serve as a key biomarker for this transformation (Chang et al., 2023). Consequently, it is hypothesized that H_2_ therapy, in conjunction with combined medication, may inhibit EndMT via the APP/CD74 pathway. This mechanism could explain the observed increase in tissue concentrations of tetrandrine following hydrogen administration.

Our study has some limitations. Firstly, we did not perform the power calculation, the small sample size may restrict the statistical significance and universality of the results. Additionally, the absence of long-term clinical validation analysis hinders a comprehensive assessment of the efficacy and safety of the combined treatment of H_2_ and Tet in a clinical setting. Therefore, future studies should aim to increase the sample size and conduct multi-center clinical trials to validate the observed results. Regarding the safety of H_2_ use, although in most of the existing studies, no severe adverse reactions directly caused by H_2_ have been reported, H_2_ is a natural product of anaerobic bacteria metabolism in the intestines. It remains to be investigated whether inhaling exogenous H_2_ would alter the H_2_ pressure in the intestines, thereby non-targetedly affecting the composition and function of the intestinal microbiota. Also, it is not clear whether long-term and high-dose use of H_2_ would interfere with certain normal cell signaling pathways mediated by reactive oxygen species that we do not fully understand. Therefore, in the process of H_2_’s clinical application, it is necessary to strengthen supervision and improve the application strategies.

## Conclusion

Our work demonstrated that OS can cause endothelial-mesenchymal transition by inhibiting mitochondrial autophagy and promoting apoptosis in silicosis. Furthermore, H_2_ combined with tetrandrine can inhibit the release of APP caused by macrophage OS and apoptosis, thereby suppressing CD74/CD44-mediated vascular EndMT, and improving the concentration of Tet in lung tissue and enhancing the therapeutic effect of Tet. In conclusion, this study has elucidated the factors contributing to the low concentration of Tet in lung tissue and proposed a solution involving combined administration with H_2_.

## Data Availability

The original contributions presented in the study are publicly available. The single-cell sequencing data presented in the study are deposited in the GEO repository, accession link http://www.ncbi.nlm.nih.gov/bioproject/1381286.

## References

[B1] Adamcakova J. Mokra D. (2021). New insights into pathomechanisms and treatment possibilities for lung silicosis. Int. J. Mol. Sci. 22 (8), 4162. 10.3390/ijms22084162 33920534 PMC8072896

[B2] Akgun M. Ergan B. (2018). Silicosis in Turkey: is it an endless nightmare or is there still hope? Turk Thorac. J. 19 (2), 89–93. 10.5152/turkthoracj.2018.040189 29755813 PMC5937816

[B3] Andreyev A. Y. Kushnareva Y. E. Starkov A. A. (2005). Mitochondrial metabolism of reactive oxygen species. Biochem. (Mosc) 70 (2), 200–214. 10.1007/s10541-005-0102-7 15807660

[B4] Bai L. Wang J. Wang X. Wang J. Zeng W. Pang J. (2025). Combined therapy with pirfenidone and nintedanib counteracts fibrotic silicosis in mice. Br. J. Pharmacol. 182 (5), 1143–1163. 10.1111/bph.17390 39546810

[B5] Cao Z. Song M. Liu Y. Pang J. Li Z. Qi X. (2020). A novel pathophysiological classification of silicosis models provides some new insights into the progression of the disease. Ecotoxicol. Environ. Saf. 202, 110834. 10.1016/j.ecoenv.2020.110834 32622305

[B6] Chan E. W. C. Wong S. K. Chan H. T. (2021). An overview on the chemistry, pharmacology and anticancer properties of tetrandrine and fangchinoline (alkaloids) from Stephania tetrandra roots. J. Integr. Med. 19 (4), 311–316. 10.1016/j.joim.2021.01.001 33583757

[B7] Chang C. J. Lai Y. J. Tung Y. C. Wu L. S. Hsu L. A. Tseng C. N. (2023). Osteopontin mediation of disturbed flow-induced endothelial mesenchymal transition through CD44 is a novel mechanism of neointimal hyperplasia in arteriovenous fistulae for hemodialysis access. Kidney Int. 103 (4), 702–718. 10.1016/j.kint.2022.12.022 36646166

[B8] Greenberg M. I. Waksman J. Curtis J. (2007). Silicosis: a review. Dis. Mon. 53 (8), 394–416. 10.1016/j.disamonth.2007.09.020 17976433

[B9] Hoy R. F. Chambers D. C. (2020). Silica-related diseases in the modern world. Allergy 75 (11), 2805–2817. 10.1111/all.14202 31989662

[B10] Huang H. Chen M. Liu F. Wu H. Wang J. Chen J. (2019). N-acetylcysteine tiherapeutically protects against pulmonary fibrosis in a mouse model of silicosis. Biosci. Rep. 39 (7). 10.1042/bsr20190681 31273057 PMC6639458

[B11] Huang X. Liang R. Liu Y. Yu L. Yang M. Shang B. (2024). Incidence, mortality, and disability-adjusted life years due to silicosis worldwide, 1990-2019: evidence from the global burden of disease study 2019. Environ. Sci. Pollut. Res. Int. 31 (25), 36910–36924. 10.1007/s11356-024-33701-3 38758446

[B12] Huang W. T. Cheng T. J. Huang L. H. Hou Y. T. (2025). Efficacy of a hydrogen-oxygen generator in treating cigarette smoke-induced chronic obstructive pulmonary disease in rats. Curr. Res. Toxicol. 8, 100214. 10.1016/j.crtox.2024.100214 39839142 PMC11745982

[B13] Ikawa M. Yoneda M. (2009). Mitochondrial dysfunction as a promoting factor of senescence. Nihon Rinsho 67 (7), 1321–1325. 19591279

[B14] Jiang R. Han L. Gao Q. Chao J. (2021). ZC3H4 mediates silica-induced EndoMT via ER stress and autophagy. Environ. Toxicol. Pharmacol. 84, 103605. 10.1016/j.etap.2021.103605 33545378

[B15] Jin S. Guerrero-Juarez C. F. Zhang L. Chang I. Ramos R. Kuan C. H. (2021). Inference and analysis of cell-cell communication using CellChat. Nat. Commun. 12 (1), 1088. 10.1038/s41467-021-21246-9 33597522 PMC7889871

[B16] Langley R. J. Mishra N. C. Peña-Philippides J. C. Rice B. J. Seagrave J. C. Singh S. P. (2011). Fibrogenic and redox-related but not proinflammatory genes are upregulated in lewis rat model of chronic silicosis. J. Toxicol. Environ. Health A 74 (19), 1261–1279. 10.1080/15287394.2011.595669 21830856 PMC4058997

[B17] Leung C. C. Yu I. T. Chen W. (2012). Silicosis. Lancet 379 (9830), 2008–2018. 10.1016/s0140-6736(12)60235-9 22534002

[B18] Li Y. Bing R. Liu M. Shang Z. Huang Y. Zhou K. (2024). Can molecular hydrogen supplementation reduce exercise-induced oxidative stress in healthy adults? A systematic review and meta-analysis. Front. Nutr. 11, 1328705. 10.3389/fnut.2024.1328705 38590828 PMC10999621

[B19] Li J. J. Cui P. Jing H. Chen S. Ma L. Zhang W. (2024). Hydrogen combined with tetrandrine attenuates silica-induced pulmonary fibrosis via suppressing NF-kappaB/NLRP3 signaling pathway-mediated epithelial mesenchymal transition and inflammation. Int. Immunopharmacol. 138, 112563. 10.1016/j.intimp.2024.112563 38943976

[B20] Liang J. Yu Y. Wang B. Lu B. Zhang J. Zhang H. (2013). Ginsenoside Rb1 attenuates oxygen-glucose deprivation-induced apoptosis in SH-SY5Y cells via protection of mitochondria and inhibition of AIF and cytochrome c release. Molecules 18 (10), 12777–12792. 10.3390/molecules181012777 24135936 PMC6270437

[B21] Liu G. Liu Y. Jin L. Li C. Nie L. Wei Y. (2016). Effect of Xinjiang Uyghur Vernonia anthelmintica willd injection treatment with silicosis fibrosis. Biomed. Res. Int. 2016, 5139651. 10.1155/2016/5139651 27803925 PMC5075590

[B22] Liu G. Yu J. Li C. Zhou X. Nie L. Wei Y. (2019). Application of protein chip combined with SELDI-TOF-MS detection to investigate serum protein expression in patients with silicosis fibrosis. Exp. Ther. Med. 17 (3), 2172–2184. 10.3892/etm.2019.7166 30783481 PMC6364189

[B23] Liu S. F. Li C. L. Lee H. C. Chang H. C. Liu J. F. Kuo H. C. (2024). The benefit of hydrogen gas as an adjunctive therapy for chronic obstructive pulmonary disease. Med. Kaunas. 60 (2), 245. 10.3390/medicina60020245 38399533 PMC10890181

[B24] Ohsawa I. Ishikawa M. Takahashi K. Watanabe M. Nishimaki K. Yamagata K. (2007). Hydrogen acts as a therapeutic antioxidant by selectively reducing cytotoxic oxygen radicals. Nat. Med. 13 (6), 688–694. 10.1038/nm1577 17486089

[B25] Ohta S. (2012). Molecular hydrogen is a novel antioxidant to efficiently reduce oxidative stress with potential for the improvement of mitochondrial diseases. Biochim. Biophys. Acta 1820 (5), 586–594. 10.1016/j.bbagen.2011.05.006 21621588

[B26] Rosengarten D. Fox B. D. Fireman E. Blanc P. D. Rusanov V. Fruchter O. (2017). Survival following lung transplantation for artificial stone silicosis relative to idiopathic pulmonary fibrosis. Am. J. Ind. Med. 60 (3), 248–254. 10.1002/ajim.22687 28145560

[B27] Song M. Y. Wang J. x. Sun Y. l. Han Z. f. Zhou Y. t. Liu Y. (2022). Tetrandrine alleviates silicosis by inhibiting canonical and non-canonical NLRP3 inflammasome activation in lung macrophages. Acta Pharmacol. Sin. 43 (5), 1274–1284. 10.1038/s41401-021-00693-6 34417574 PMC9061833

[B28] Szeto H. H. (2006). Mitochondria-targeted peptide antioxidants: novel neuroprotective agents. AAPS J. 8 (3), E521–E531. 10.1208/aapsj080362 17025271 PMC2761060

[B29] Tian Y. Shi H. Zhang D. Wang C. Zhao F. Li L. (2023). Nebulized inhalation of LPAE-HDAC10 inhibits acetylation-mediated ROS/NF-κB pathway for silicosis treatment. J. Control Release 364, 618–631. 10.1016/j.jconrel.2023.10.018 37848136

[B30] Wang L. Zhao M. Qian R. Wang M. Bao Q. Chen X. (2022). Nicotinamide mononucleotide ameliorates silica-induced lung injury through the Nrf2-Regulated glutathione metabolism pathway in mice. Nutrients 15 (1), 143. 10.3390/nu15010143 36615800 PMC9823503

[B31] Wei Y. Sun L. Liu C. Li L. (2023). Naringin regulates endoplasmic reticulum stress and mitophagy through the ATF3/PINK1 signaling axis to alleviate pulmonary fibrosis. Naunyn Schmiedeb. Arch. Pharmacol. 396 (6), 1155–1169. 10.1007/s00210-023-02390-z 36688958

[B32] Yang H. J. Tsou W. H. Shen M. C. Liu C. Y. Saunders H. M. Wang K. Y. (2022). The effects of hydrogen treatment in a cigarette smoke solution-induced chronic obstructive pulmonary disease-like changes in an animal model. J. Thorac. Dis. 14 (11), 4246–4255. 10.21037/jtd-22-324 36524091 PMC9745525

[B33] Yildiz F. LeBaron T. W. Alwazeer D. (2025). A comprehensive review of molecular hydrogen as a novel nutrition therapy in relieving oxidative stress and diseases: mechanisms and perspectives. Biochem. Biophys. Rep. 41, 101933. 10.1016/j.bbrep.2025.101933 39911528 PMC11795818

[B34] Yu X. Y. Song Y. H. Geng Y. J. Lin Q. X. Shan Z. X. Lin S. G. (2008). Glucose induces apoptosis of cardiomyocytes via microRNA-1 and IGF-1. Biochem. Biophys. Res. Commun. 376 (3), 548–552. 10.1016/j.bbrc.2008.09.025 18801338

[B35] Zhang H. Li Q. Yao R. Guo N. (1997). Experimental studies on the therapeutic effects of lung lavage with large volume of saline on silicosis. Wei Sheng Yan Jiu 26 (2), 77–79. 10325605

[B36] Zhang E. Yang Y. Chen S. Peng C. Lavin M. F. Yeo A. J. (2018). Bone marrow mesenchymal stromal cells attenuate silica-induced pulmonary fibrosis potentially by attenuating Wnt/β-catenin signaling in rats. Stem Cell Res. Ther. 9 (1), 311. 10.1186/s13287-018-1045-4 30428918 PMC6234553

[B37] Zhang C. Xing Y. Wu X. Jiang Q. Luo X. He W. (2024). Inhalation of hydrogen gas protects against mitomycin-induced pulmonary veno-occlusive disease. Respir. Res. 25 (1), 281. 10.1186/s12931-024-02906-y 39014440 PMC11253336

[B38] Zhao Y. Xu G. Li H. Chang M. Guan Y. Li Y. (2020). Overexpression of endogenous lipoic acid synthase attenuates pulmonary fibrosis induced by crystalline silica in mice. Toxicol. Lett. 323, 57–66. 10.1016/j.toxlet.2020.01.023 32017981

