# Supplementary figures and images for: Hydrogen improves the efficacy of tetrandrine in the treatment of silicosis by inhibiting vascular endothelial mesenchymal transition caused by oxidative stress
Source: Front Bioeng Biotechnol. 2026 Jan 5;13:1668524. doi: 10.3389/fbioe.2025.1668524 (PMC12813091; doi:10.3389/fbioe.2025.1668524)

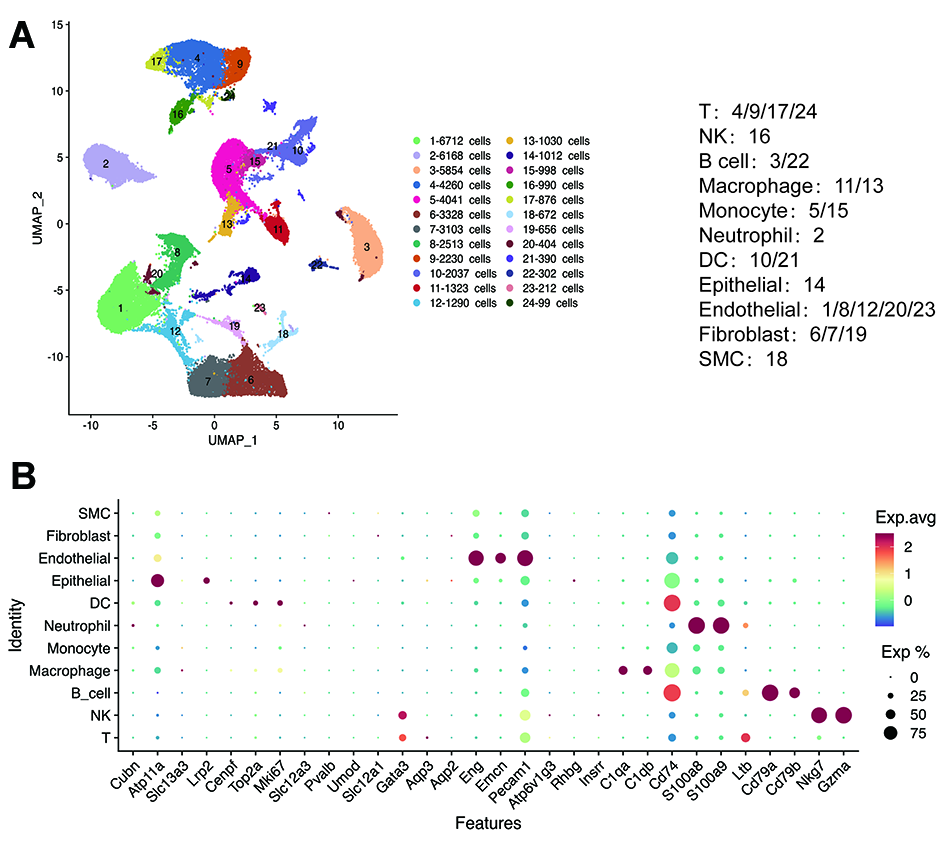

Supplement: Supplementary file 1 [file Image3.tif]

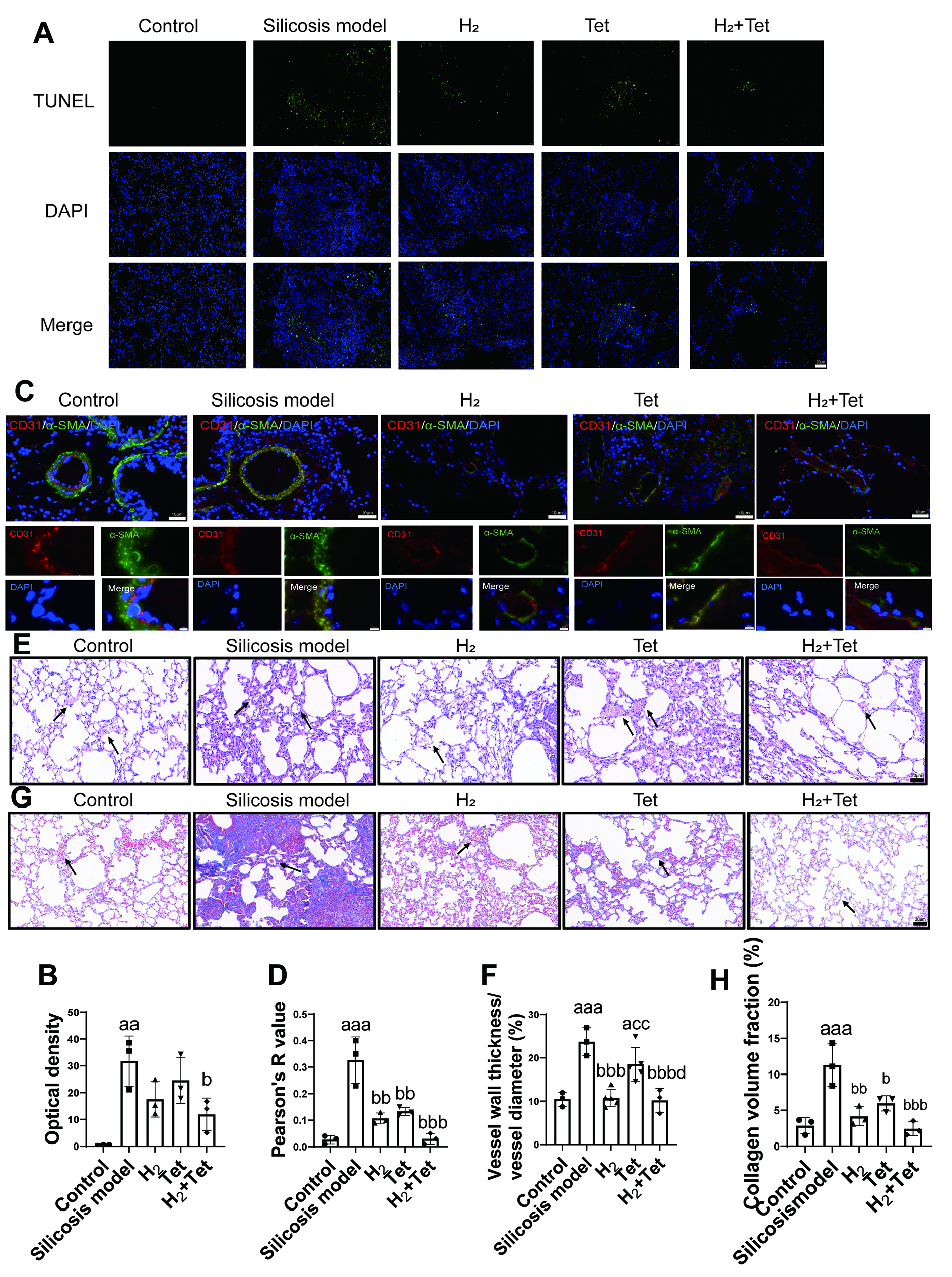

Supplement: Supplementary file 2 [file Image2.tif]

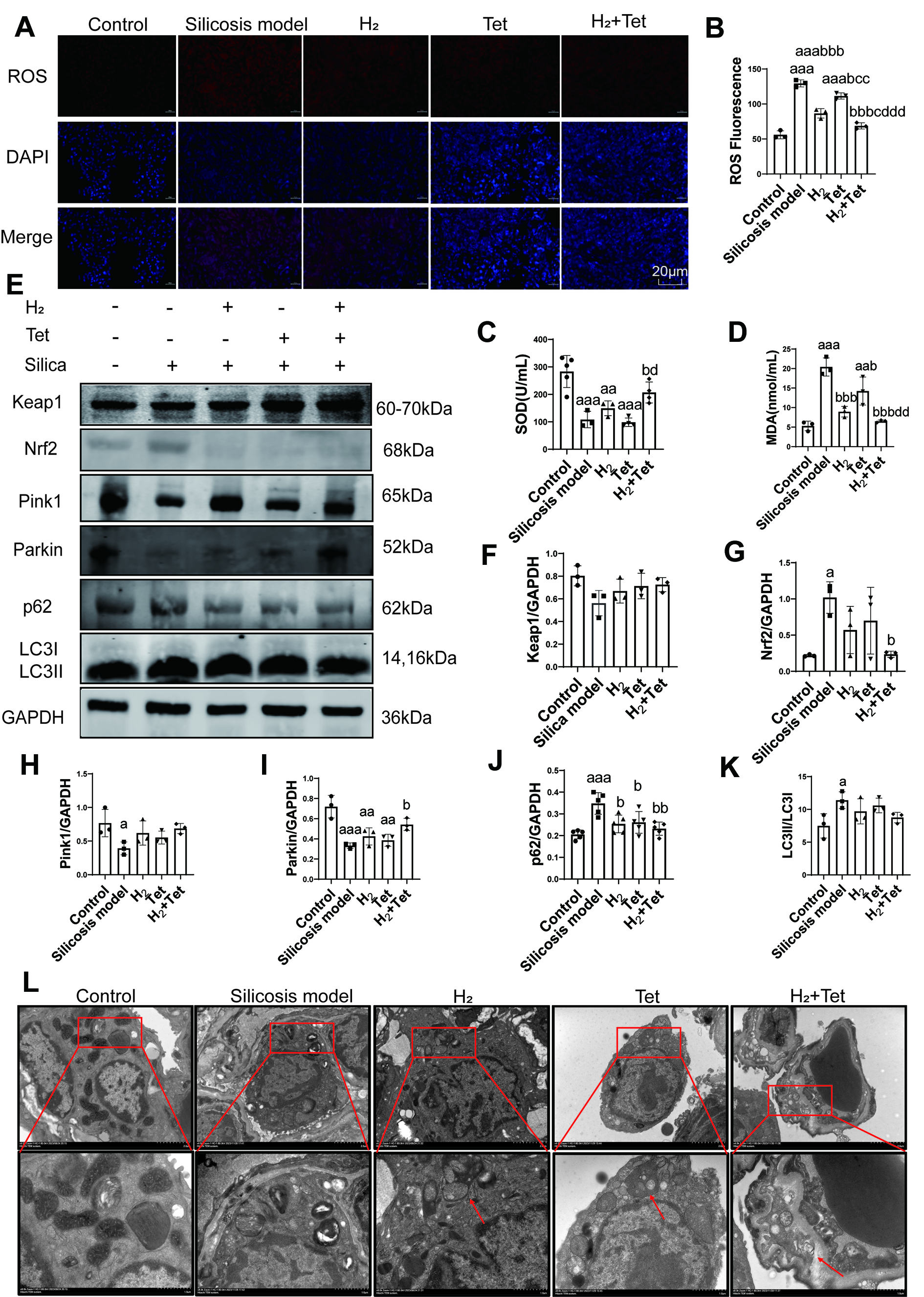

Supplement: Supplementary file 3 [file Image1.tif]
